# Decreased Monocyte HLA-DR Expression in Patients with Sepsis and Acute Kidney Injury

**DOI:** 10.3390/medicina58091198

**Published:** 2022-09-01

**Authors:** Huang-Pin Wu, Li-Pang Chuang, Pi-Hua Liu, Chien-Ming Chu, Chung-Chieh Yu, Shih-Wei Lin, Kuo-Chin Kao, Li-Fu Li, Duen-Yau Chuang

**Affiliations:** 1 Division of Pulmonary, Critical Care and Sleep Medicine, Chang Gung Memorial Hospital, Keelung 20401, Taiwan; 2 College of Medicine, Chang Gung University, Taoyuan 33302, Taiwan; 3 Department of Pulmonary and Critical Care Medicine, Linkou Chang Gung Memorial Hospital, Taoyuan 33305, Taiwan; 4 Clinical Informatics and Medical Statistics Research Center, College of Medicine, Chang Gung University, Taoyuan 33302, Taiwan; 5 Division of Endocrinology and Metabolism, Department of Internal Medicine, Linkou Chang Gung Memorial Hospital, Taoyuan 33305, Taiwan; 6 Department of Chemistry, National Chung-Hsing University, Taichung 40227, Taiwan

**Keywords:** monocyte, HLA-DR, acute kidney injury, sepsis

## Abstract

*Background and objectives:* Acute kidney injury (AKI) is common in critically ill patients, especially those with sepsis. Persistently low human leukocyte antigen (HLA)-DR expression in monocytes reflects the decreased function of antigen-presenting cells, contributing to poor outcomes in sepsis. This study aimed to establish an association between AKI and HLA-DR expression in monocytes of patients with sepsis. *Materials and Methods:* We detected HLA-DR expression in monocytes and measured plasma levels of S100A12, high-mobility group box 1 (HMGB1), advanced glycation end products (AGE), and soluble receptor for AGE (sRAGE) from septic patients and healthy controls. *Results:* HLA-DR expression in monocytes was decreased in patients with AKI than in those without AKI (29.8 ± 5.0% vs. 53.1 ± 5.8%, *p* = 0.005). Compared with AKI patients, the mean monocyte HLA-DR expression in patients with end-stage renal disease was increased without statistical significance. There were no differences in the AGE/sRAGE ratio and plasma levels of S100A12, HMGB1, AGE, and sRAGE between patients with and without AKI. *Conclusions*: Compared with septic patients without AKI, patients with AKI had significantly lower HLA-DR expression in monocytes. The role of hemodialysis in monocyte HLA-DR expression needs further studies to explore.

## 1. Introduction

Acute kidney injury (AKI) is a common clinical disease especially in critically ill patients. AKI is also significantly associated with mortality, length of stay and healthcare costs [1]. The most common cause of AKI is sepsis [2,3]. The 3rd International Consensus Definitions for Sepsis and Septic Shock (Sepsis-3) revised the definition of sepsis as a life-threatening organ dysfunction caused by a dysregulated host response to infection [4]. Sepsis has several characteristics including biochemical, pathological, and physiological abnormalities.

The human leukocyte antigen (HLA)-DR expression reflects the function of antigen-presenting cells and monocytes. One of the factors contributing to poor outcomes in sepsis is decreased monocyte function with low expression of HLA-DR [5,6,7]. In a study enrolling patients in the intensive care unit (ICU), Day 1 HLA-DR expression in monocytes of patients with AKI was similar to those without AKI [8]. However, this study enrolled less than 20% of patients with sepsis. To the best of our knowledge, no study has reported the relationship between AKI and HLA-DR expression in monocytes of patients with sepsis. It is unclear whether AKI is associated with decreased monocyte HLA-DR expression.

Recently, receptor for advanced glycation end products (RAGE) has been found to participate in AKI in sepsis [9]. RAGE uses its three-dimensional structure to identify different ligands. The possible ligands contain S100 proteins, advanced glycation end products (AGE), and high-mobility group box 1 (HMGB1) [10]. The isoforms of soluble RAGE play a role of decoy receptors and disturb the binding of membrane RAGE ligand. Therefore, in circulation, it is bound by soluble RAGE (sRAGE). This action avoids the combination of S100, HMGB1, or AGE with RAGE, resulting in further kidney injury.

It should be determined whether monocyte HLA-DR expression and plasma mediators are associated with the development of AKI in patients with sepsis. Hence, the aim of this prospective observational study aimed to determine the roles of HLA-DR expression and plasma mediators in septic AKI patients by blood sampling.

## 2. Materials and Methods

### 2.1. Participants

Patients admitted to medical ICU in our hospital due to sepsis were enrolled between August 2016 and July 2018. Twenty-seven healthy controls were enrolled to validate the experimental findings and they were from our health evaluation center for health examinations. Past medical histories and comorbidities were recorded. Adverse events were noted within this ICU admission in the first three days.

All patients accepted standard managements according to the guidelines [11]. This study was approved by Institutional Review Board at Chang Gung Memorial Hospital with No.103-7093B and No.104-8013C. Informed consent forms were provided by the patients’ family members. The standard inclusion criteria were used for diagnosis of sepsis and septic shock [4]. Patients younger than 18 years old, pregnant women, or patients with human immunodeficiency virus infection, referral from other hospitals or ICU, and less than 48 h between ICU admission and blood sample were excluded.

### 2.2. Definitions

A suspected/documented infection combined with a rapid increase of ≥2 points in the sequential organ failure assessment (SOFA) score defined sepsis. Low blood pressure that was not responsive to adequate resuscitation with fluid and needed vasopressors to keep mean arterial blood pressure more than 65 mmHg is considered hypotension. The definition of septic shock was a hypotension with serum level of lactate more than 18 mg/dL. Stages 1, 2, and 3 of Kidney Disease Improving Global Outcomes (KDIGO) guidelines were used to define AKI [12]. A platelet counts less than 150,000/μL defined thrombocytopenia, whereas total bilirubin more than 2 mg/dL defined jaundice. The Acute Physiology and Chronic Health Evaluation (APACHE) II score [13] and SOFA score [4] were assessed to evaluate disease severity. All adverse events were recorded within one day after ICU admission. After ICU admission, patients who survived less than 28 days were defined as non-survivors.

### 2.3. Preparation of Plasma and Peripheral Blood Mononuclear Cell (PBMC)

After ICU admission, 10 mL of whole blood was sampled from patients at 08:00–08:30 a.m. within 48 h. For controls, whole blood was sampled at 08:00–08:30 a.m. The blood was mixed with heparin to prevent blood clotting. In total, 2 mL blood was used for plasma preparation. After receiving plasma, it was immediately stored at −80 °C.

Using Ficoll-Plaque (Amersham Biosciences, Uppsala, Sweden), PBMCs were isolated from the residual 8 mL blood within 2 h of collection by way of differential centrifugation.

### 2.4. Plasma Cytokine Level Measurement

Plasma AGE levels were measured over human enzyme-linked immunosorbent assay (ELISA) kit (Cell Biolabs, Inc., San Diego, CA, USA). Plasma sRAGE and S100A12 levels were measured over human ELISA kits (R&D Systems, Inc., Minneapolis, MN, USA). Plasma HMGB1 levels were measured over human ELISA kit (MyBioSource, Inc., San Diego, CA, USA).

### 2.5. Detection of Monocyte HLA-DR Expression

A number of 5 × 10^5^ cells of PBMCs were suspended in phosphate-buffered saline (PBS) (50 μL) and incubated in the dark at room temperature for 15 min with 10 μL antibodies of CD14_APC-750_, CD11b_PC7_, and HLA-DR_FITC_ (Beckman Coulter, Brea, CA, USA). Then, the cells were re-suspended in 500 μL PBS. An eight-color flow cytofluorimeter (Beckman Coulter, Brea, CA, USA) was used to detect the HLA-DR expression in monocytes, as described in our previous study protocol [14].

### 2.6. Statistical Analysis

The Statistical Package for the Social Sciences (SPSS) software V26.0 for Mac (IBM Inc., Armonk, NY, USA) was used for statistical analysis. The independent-samples *t*-test was used to analyze differences in continuous variables between the two groups, whereas the chi-square test or Fisher’s exact test was used to analyze differences in categorical variables. Using general linear model, the association between HLA-DR expression in monocytes and all variables was analyzed. Statistically significant variables were entered into a multiple general linear model to evaluate their independent contribution to HLA-DR expression in monocytes. A *p* <0.05 was set as statistical significance.

## 3. Results

Of the 44 septic patients enrolled, 19 did not have AKI, and 17 had AKI initially. Eight patients had a history of end-stage renal disease (ESRD) and underwent regular hemodialysis. Patients’ clinical characteristics are presented in Table 1. The serum creatinine level and age of the controls was lower than that of septic patients. There were no differences in gender, age, history, infection sources, adverse events, and body weight among patients with no AKI, AKI, or ESRD. Compared with patients with AKI and ESRD, patients without AKI had lower APACHE II and SOFA scores. The 28-day mortality and serum creatinine level in patients with AKI and ESRD was higher than that in patients without AKI. Compared with patients with ESRD, patients with and without AKI had higher urine output.

### 3.1. HLA-DR Expression in Monocytes and Plasma AGE, sRAGE, HMGB1 and S100A12 Levels in Controls and Septic Patients

**Figure 1:**
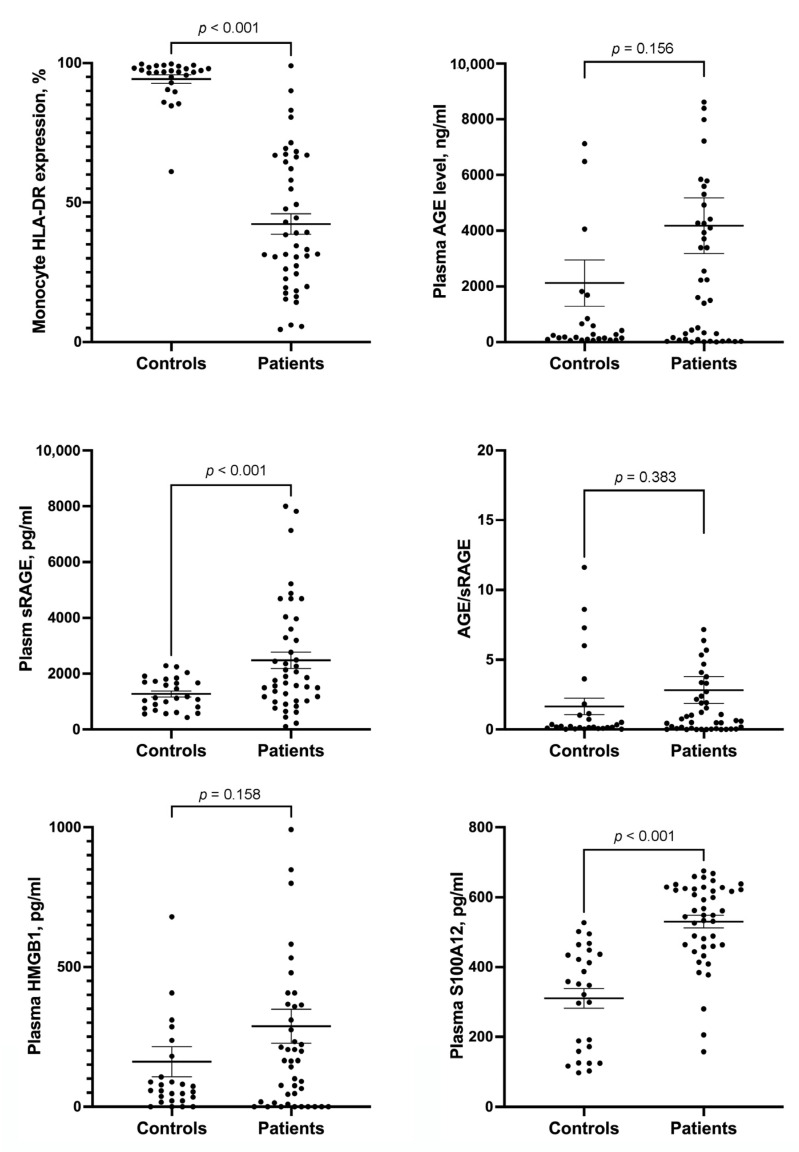
Scatterplots showing the levels of monocyte HLA-DR expression, plasma AGE, plasma sRAGE, AGE/sRAGE ratio, plasma HMGB1, and plasma S100A12 between controls and patients with sepsis. Error bar represents mean and standard error mean. HLA = human leukocyte antigen; AGE = advanced glycation end products; sRAGE = soluble receptor for AGE; HMGB1 = high-mobility group box 1.

HLA-DR expression in monocytes of septic patients was lower than that in the controls (Figure 1). Compared with septic patients, the controls had lower plasma sRAGE and S100A12 levels. The AGE/sRAGE ratio and plasma levels of HMBG1 and AGE were similar between the controls and septic patients.

### 3.2. HLA-DR Expression in Monocytes and Plasma AGE, sRAGE, HMGB1 and S100A12 Levels in Patients with No AKI, AKI, and ESRD

**Figure 2:**
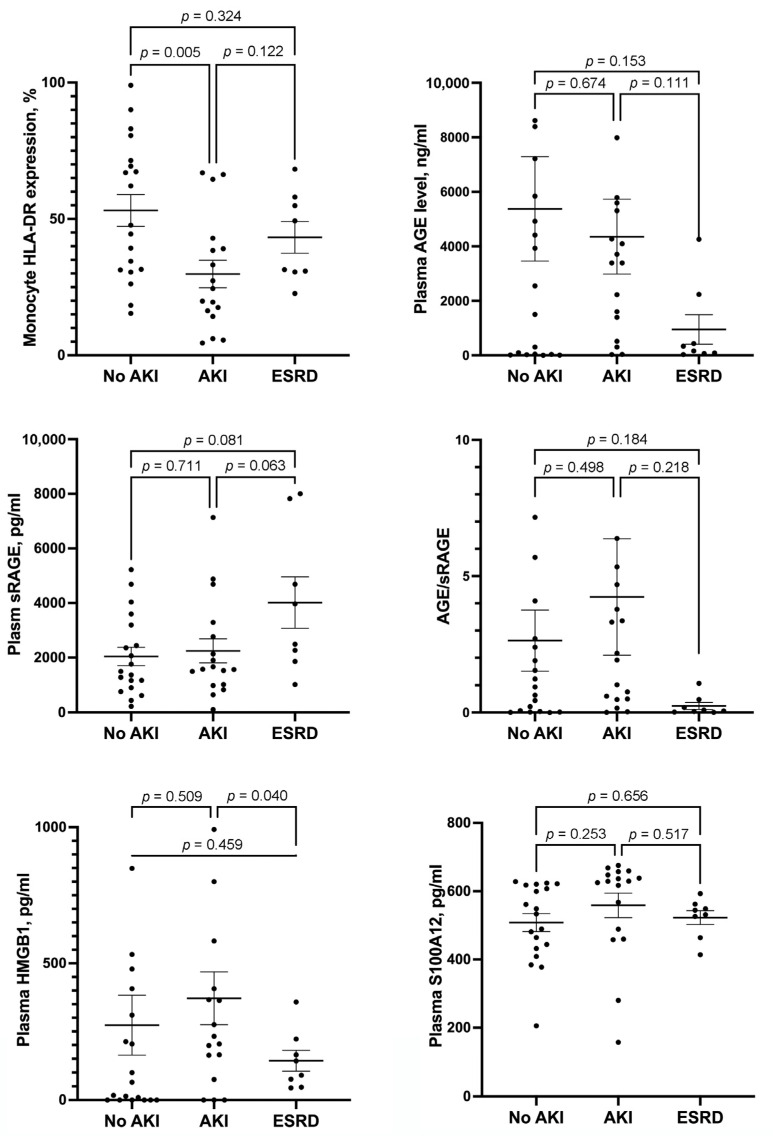
Scatterplots showing the levels of monocyte HLA-DR expression, plasma AGE, plasma sRAGE, AGE/sRAGE ratio, plasma HMGB1, and plasma S100A12 among septic patients with and without AKI and those with ESRD. Error bar represents mean and standard error mean. HLA = human leukocyte antigen; AGE = advanced glycation end products; sRAGE = soluble receptor for AGE; HMGB1 = high-mobility group box 1; AKI = acute kidney injury; ESRD = end stage renal disease.

HLA-DR expression in monocytes was significantly lower in patients with AKI than in those without AKI (Figure 2). HLA-DR expression in monocytes of patients with ESRD was similar to that of patients with and without AKI. There were no differences in the AGE/sRAGE ratio and plasma levels of S100A12, sRAGE, and AGE among patients with and without AKI and those with ESRD. Plasma HMGB1 level was significantly lower in patients with ESRD than in those with AKI. Plasma HMGB1 level in patients without AKI was similar to that of patients with AKI and ESRD.

### 3.3. Effect of AKI on HLA-DR Expression in Monocytes

According to the univariate analyses, 28-day mortality and the presence of AKI and GI bleeding were associated with HLA-DR expression in monocytes (Table 2). Multiple general linear model analysis found that AKI (odds ratio, −18.425; 95% confidence interval, −35.278 to −1.573) was still negatively independently associated with HLA-DR expression in monocytes.

## 4. Discussion

We first demonstrated that HLA-DR expression in monocytes of septic patients with AKI was lower than in those without AKI. HLA-DR expression in monocytes was similar between patients with ESRD and no AKI. This suggests that certain unknown mediators that decrease HLA-DR expression in monocytes might be partially eliminated by hemodialysis. The sequential measurement of HLA-DR expression in monocytes has gained much interest over the past 20 years to identify immune alterations in critically ill patients [15]. No recovery from serial circulating monocyte HLA-DR expression has been associated with the high mortality in patients with sepsis [6,16]. It is reasonable to hypothesize that patients with sepsis and no improvement in renal function from AKI would be persistently with low expression of monocyte HLA-DR, which might result in immune depression and increased risk of mortality.

The exact mechanism between AKI and decreased monocyte HLA-DR expression remains unclear. One of the pathogeneses of sepsis-induced AKI is oxidative stress, which was induced by systemic and intrarenal generation of reactive oxygen species [17]. Oxidative stress can directly exert renal parenchymal damage and may intensify renal microvascular and functional dysregulation. Since patients with sepsis and AKI had higher oxidative stress and lower monocyte HLA-DR expression than healthy subjects [18], high oxidative stress might be one of the possible mechanisms resulting in decreased monocyte HLA-DR expression in patients with sepsis and AKI.

In this study, plasma levels of AGE, sRAGE and AGE to sRAGE ratio were similar between septic patients with and without AKI. In contrast to the study by Helena et al., plasma sRAGE levels were higher in septic patients with AKI than in those without AKI [19]. Recently, Taro et al. found that the addition of recombinant sRAGE canceled hypoxia-induced inflammation and promoted cell viability in cultured murine tubular epithelial cells [20]. sRAGE administration might prevent renal tubular damage in ischemia/reperfusion-induced AKI models. After searching the PubMed database, no study reported the difference in the ratio of AGE to sRAGE between septic patients with and without AKI. However, serum AGE to sRAGE ratio levels were 313% higher in patients with ESRD than in control subjects [21]. The roles of sRAGE and the AGE to sRAGE ratio in AKI in septic patients require further studies.

In a cross-sectional study enrolling patients without sepsis, serum S100A12 and HMGB1 levels were elevated in patients with AKI compared to those in patients on hemodialysis [22]. Within 7 days of cardiac surgery, patients who developed AKI had higher plasma S100A12 levels [23]. In our study, there was no difference in plasma S100A12 levels among septic patients with and without AKI and those with ESRD. More studies are needed to clarify the role of S100A12 in sepsis-induced AKI. HMGB1 is a damage-associated molecular pattern (DAMP), which is a group of immunostimulatory molecules that participate in the inflammatory response after tissue injury [24]. Sepsis induces renal tubular necrosis with the release of DAMPs, leading to AKI. However, the relative contribution of different DAMPs to renal dysfunction remains unclear. Studies have found that silent information regulator 2-related enzyme 1 and glutamine could ameliorate sepsis-induced AKI by influencing HMGB1 deacetylation or downregulating the HMBG1-mediated pathway [25,26]. In this study, the association between high plasma HMGB1 levels and AKI development was not identified. These results suggest that HMGB1 has a lower contribution to AKI in patients with sepsis.

Our study has a major limitation. We enrolled a relatively small number of patients with sepsis. This resulted in a patient number lower than 10 in the ESRD group, which might result in no statistical difference in monocyte HLA-RD expression and plasma levels of AGE, sRAGE, AGE to sRAGE ratio, and HMBG1 between patients with AKI and ESRD. Furthermore, there are three AKI stages in the KDIGO clinical practice guidelines. In this study, the number of patients was too small to divide into three AKI groups based on three AKI stages in the KDIGO clinical practice guidelines. It is possible that monocyte HLA-DR expression was different among the three AKI stages and lowest in septic patients with AKI stage 3. A large-scale study is required to confirm this.

## 5. Conclusions

Compared with septic patients without AKI, patients with AKI had significantly lower monocyte HLA-DR expression. Compared with the AKI group, the mean monocyte HLA-DR expression in the ESRD group was increased, even without statistical significance. The role of hemodialysis in monocyte HLA-DR expression needs further studies to explore.

## Figures and Tables

**Table 1:** Clinical characteristics in controls and patients with sepsis (number, mean ± standard error mean).

	No AKI (*n* = 19)	AKI (*n* = 17)	ESRD (*n* = 8)	All Patients (*n* = 44)	Controls (*n* = 27)
Age (years)	76.3 ± 2.6	73.9 ± 2.5	77.0 ± 3.6	75.5 ± 1.6	60.3 ± 1.3 *
Male (%)	14 (73.7)	10 (58.8)	2 (25.0)	26 (59.1)	17 (63.0)
APACHE II score	18.0 ± 0.9	26.9 ± 1.0 ^†^	27.8 ± 3.3 ^†^	23.2 ± 1.1	
SOFA score	8.5 ± 0.7	11.9 ± 0.9 ^†^	12.3 ± 0.8 ^†^	10.5 ± 0.6	
History (%)					
COPD	1 (5.3)	1 (5.9)	0 (0.0)	2 (4.5)	
Heart failure	3 (15.8)	2 (11.8)	0 (0.0)	5 (11.4)	
Hypertension	14 (73.7)	9 (52.9)	7 (87.5)	30 (68.2)	
Diabetes mellitus	7 (36.8)	6 (35.3)	6 (75.0)	19 (43.2)	
Old CVA	5 (26.3)	4 (23.5)	1 (12.5)	10 (22.7)	
Liver cirrhosis	2 (10.5)	3 (17.6)	0 (0.0)	5 (11.4)	
Active malignancy	1 (5.3)	1 (5.9)	0 (0.0)	2 (4.5)	
Infection source					
Pneumonia	13 (68.4)	13 (76.5)	3 (37.5)	29 (65.9)	
UTI	3 (15.8)	0 (0.0)	2 (25.0)	5 (11.4)	
Others	3 (15.8)	4 (23.5)	3 (37.5)	10 (22.7)	
Adverse event					
New arrhythmia	3 (15.8)	1 (5.9)	0 (0.0)	4 (9.1)	
GI bleeding	1 (5.3)	3 (17.6)	0 (0.0)	4 (9.1)	
Shock	8 (42.1)	11 (64.7)	6 (75.0)	25 (56.8)	
Thrombocytopenia	5 (26.3)	7 (41.2)	3 (37.5)	15 (34.1)	
Jaundice	3 (15.8)	5 (29.4)	0 (0.0)	8 (18.2)	
Bacteremia	3 (15.8)	2 (11.8)	2 (25.0)	7 (15.9)	
Body weight, kg	62.5 ± 3.6	60.6 ± 3.7	52.0 ± 4.3	59.9 ± 2.3	65.5 ± 1.9
Serum creatinine, mg/dL	1.25 ± 0.18	3.32 ± 0.36 ^†^	6.25 ± 1.31^†^	2.96 ± 0.39	0.88 ± 0.05 *
Urine output, mL/kg/h	0.97 ± 0.14	0.72 ± 0.17	0.05 ± 0.03 ^†,‡^	0.70 ± 0.10	
28-day mortality	1 (5.3)	9 (52.9) ^†^	4 (50.0) ^†^	14 (31.8)	

Abbreviations: AKI = acute kidney injury; ESRD = end-stage renal disease; APACHE = Acute Physiology and Chronic Health Evaluation; SOFA = Sequential Organ Failure Assessment; COPD = chronic obstructive pulmonary disease; CVA = cerebral vascular accident; UTI = urinary tract infection; GI = gastrointestinal. * *p* < 0.05, comparison with patients using *t*-test. ^†^ *p* < 0.05, comparison with no AKI group using *t*-test or Fisher’s exact test. ^‡^ *p* < 0.05, comparison with AKI group using *t*-test.

**Table 2:** General linear model to analyze the independent factors for HLA-DR expression in monocytes of septic patients.

Variables	Univariable B (95% CI)	*p* Value	Multivariable B (95% CI)	*p* Value
28-day mortality	−17.018 (−32.141 to −1.894)	0.028	−5.689 (−22.953 to 11.575)	0.509
AKI	−23.289 (−38.329 to −8.248)	0.003	−18.425 (−35.278 to −1.573)	0.033
ESRD	−9.862 (−28.850 to 9.126)	0.300	−8.231 (−28.692 to 12.229)	0.421
GI bleeding	−25.795 (−50.484 to −1.106)	0.041	−17.369 (−42.396 to 7.658)	0.168

Abbreviations: HLA = human leukocyte antigen; CI = confidence interval; AKI = acute kidney injury; ESRD = end-stage renal disease; GI = gastrointestinal.

## Data Availability

The datasets used during this study are available from the corresponding author upon reasonable request.

## References

[1] Chertow G.M., Burdick E., Honour M., Bonventre J.V., Bates D.W. (2005). Acute kidney injury, mortality, length of stay, and costs in hospitalized patients. J. Am. Soc. Nephrol..

[2] Nakano D. (2020). Septic acute kidney injury: A review of basic research. Clin. Exp. Nephrol..

[3] Rossaint J., Zarbock A. (2016). Acute kidney injury: Definition, diagnosis and epidemiology. Minerva Urol. Nephrol..

[4] Singer M., Deutschman C.S., Seymour C.W., Shankar-Hari M., Annane D., Bauer M., Bellomo R., Bernard G.R., Chiche J.D., Coopersmith C.M. (2016). The Third International Consensus Definitions for Sepsis and Septic Shock (Sepsis-3). JAMA.

[5] Lukaszewicz A.C., Grienay M., Resche-Rigon M., Pirracchio R., Faivre V., Boval B., Payen D. (2009). Monocytic HLA-DR expression in intensive care patients: Interest for prognosis and secondary infection prediction. Crit. Care Med..

[6] Wu H.P., Shih C.C., Lin C.Y., Hua C.C., Chuang D.Y. (2011). Serial increase of IL-12 response and human leukocyte antigen-DR expression in severe sepsis survivors. Crit. Care.

[7] Leijte G.P., Rimmele T., Kox M., Bruse N., Monard C., Gossez M., Monneret G., Pickkers P., Venet F. (2020). Monocytic HLA-DR expression kinetics in septic shock patients with different pathogens, sites of infection and adverse outcomes. Crit. Care.

[8] Ahlstrom A., Hynninen M., Tallgren M., Kuusela P., Valtonen M., Orko R., Siitonen S., Takkunen O., Pettila V. (2004). Predictive value of interleukins 6, 8 and 10, and low HLA-DR expression in acute renal failure. Clin. Nephrol..

[9] Fukami K., Taguchi K., Yamagishi S., Okuda S. (2015). Receptor for advanced glycation endproducts and progressive kidney disease. Curr. Opin. Nephrol. Hypertens..

[10] Leclerc E., Fritz G., Vetter S.W., Heizmann C.W. (2009). Binding of S100 proteins to RAGE: An update. Biochim. Biophys. Acta.

[11] Rhodes A., Evans L.E., Alhazzani W., Levy M.M., Antonelli M., Ferrer R., Kumar A., Sevransky J.E., Sprung C.L., Nunnally M.E. (2017). Surviving Sepsis Campaign: International Guidelines for Management of Sepsis and Septic Shock: 2016. Intensive Care Med..

[12] Khwaja A. (2012). KDIGO clinical practice guidelines for acute kidney injury. Nephron Clin. Pract..

[13] Knaus W.A., Draper E.A., Wagner D.P., Zimmerman J.E. (1985). APACHE II: A severity of disease classification system. Crit. Care Med..

[14] Wu H.P., Lin Y.K. (2018). Effect of *Eucommia ulmoides* Oliv., *Gynostemma pentaphyllum* (Thunb.) Makino, and *Curcuma longa* L. on Th1- and Th2-cytokine responses and human leukocyte antigen-DR expression in peripheral blood mononuclear cells of septic patients. J. Ethnopharmacol..

[15] Zhuang Y., Peng H., Chen Y., Zhou S., Chen Y. (2017). Dynamic monitoring of monocyte HLA-DR expression for the diagnosis, prognosis, and prediction of sepsis. Front. Biosci. Landmark.

[16] Wu J.F., Ma J., Chen J., Ou-Yang B., Chen M.Y., Li L.F., Liu Y.J., Lin A.H., Guan X.D. (2011). Changes of monocyte human leukocyte antigen-DR expression as a reliable predictor of mortality in severe sepsis. Crit. Care.

[17] Heyman S.N., Rosen S., Rosenberger C. (2011). A role for oxidative stress. Contrib. Nephrol..

[18] Silva S., de Cal M., Cruz D., Lentini P., Corradi V., Gallo G., Salvatori G., Verbine A., Pogoshyan L., Nalesso F. (2008). Oxidative stress and ‘monocyte reprogramming’ in septic patients with acute kidney injury requiring CRRT. Blood Purif..

[19] Brodska H., Malickova K., Valenta J., Fabio A., Drabek T. (2013). Soluble receptor for advanced glycation end products predicts 28-day mortality in critically ill patients with sepsis. Scand. J. Clin. Lab. Investig..

[20] Miyagawa T., Iwata Y., Oshima M., Ogura H., Sato K., Nakagawa S., Yamamura Y., Kamikawa Y., Miyake T., Kitajima S. (2021). Soluble receptor for advanced glycation end products protects from ischemia- and reperfusion-induced acute kidney injury. Biol. Open.

[21] Prasad K. (2019). Is there any evidence that AGE/sRAGE is a universal biomarker/risk marker for diseases?. Mol. Cell Biochem..

[22] Zakiyanov O., Kriha V., Vachek J., Zima T., Tesar V., Kalousova M. (2013). Placental growth factor, pregnancy-associated plasma protein-A, soluble receptor for advanced glycation end products, extracellular newly identified receptor for receptor for advanced glycation end products binding protein and high mobility group box 1 levels in patients with acute kidney injury: A cross sectional study. BMC Nephrol..

[23] Nikolakopoulou Z., Hector L.R., Creagh-Brown B.C., Evans T.W., Quinlan G.J., Burke-Gaffney A. (2019). Plasma S100A8/A9 heterodimer is an early prognostic marker of acute kidney injury associated with cardiac surgery. Biomark. Med..

[24] Ludes P.O., de Roquetaillade C., Chousterman B.G., Pottecher J., Mebazaa A. (2021). Role of Damage-Associated Molecular Patterns in Septic Acute Kidney Injury, from Injury to Recovery. Front. Immunol..

[25] Wei S., Gao Y., Dai X., Fu W., Cai S., Fang H., Zeng Z., Chen Z. (2019). SIRT1-mediated HMGB1 deacetylation suppresses sepsis-associated acute kidney injury. Am. J. Physiol. Renal Physiol..

[26] Hu Y.M., Pai M.H., Yeh C.L., Hou Y.C., Yeh S.L. (2012). Glutamine administration ameliorates sepsis-induced kidney injury by downregulating the high-mobility group box protein-1-mediated pathway in mice. Am. J. Physiol. Renal Physiol..

